# Picometre-level surface control of a closed-loop, adaptive X-ray mirror with integrated real-time interferometric feedback

**DOI:** 10.1107/S1600577524011007

**Published:** 2025-01-01

**Authors:** Ioana-Theodora Nistea, Simon G. Alcock, Andrew Foster, Vivek Badami, Riccardo Signorato, Matteo Fusco

**Affiliations:** a Optics and Metrology Diamond Light Source Harwell Science and Innovation Campus DidcotOX11 0DE United Kingdom; b Observatory Sciences Ltd, United Kingdom; c Zygo Corporation, Middlefield, CT06441, USA; d S.RI.Tech, Vigonza, Italy; e CAEN, Viareggio, Italy; Tohoku University, Japan

**Keywords:** adaptive optics, X-ray optics, bimorph deformable mirrors, closed-loop controls, fibre optic interferometers, high-voltage power supplies, X-ray mirrors

## Abstract

We provide a technical description and experimental results of the practical development and offline testing of an innovative, closed-loop, adaptive mirror system capable of making rapid, precise and ultra-stable changes in the size and shape of reflected X-ray beams generated at synchrotron light and free-electron laser facilities. The optical surface of a piezoelectric bimorph deformable mirror is continuously monitored at 20 kHz by an array of interferometric sensors. This matrix of height data is autonomously converted into voltage commands that are sent at 1 Hz to the piezo actuators to modify the shape of the mirror optical surface. Hence, users can rapidly switch in closed-loop between pre-calibrated X-ray wavefronts by selecting the corresponding freeform optical profile. This closed-loop monitoring is shown to repeatably bend and stabilize the low- and mid-spatial frequency components of the mirror surface to any given profile with an error <200 pm peak-to-valley, regardless of the recent history of bending and hysteresis. Without closed-loop stabilization after bending, the mirror height profile is shown to drift by hundreds of nanometres, which will slowly distort the X-ray wavefront. The metrology frame that holds the interferometric sensors is designed to be largely insensitive to temperature changes, providing an ultra-stable reference datum to enhance repeatability. We demonstrate an unprecedented level of fast and precise optical control in the X-ray domain: the profile of a macroscopic X-ray mirror of over 0.5 m in length was freely adjusted and stabilized to atomic level height resolution. Aside from demonstrating the extreme sensitivity of the interferometer sensors, this study also highlights the voltage repeatability and stability of the programmable high-voltage power supply, the accuracy of the correction-calculation algorithms and the almost instantaneous response of the bimorph mirror to command voltage pulses. Finally, we demonstrate the robustness of the system by showing that the bimorph mirror’s optical surface was not damaged by more than 1 million voltage cycles, including no occurrence of the ‘junction effect’ or weakening of piezoelectric actuator strength. Hence, this hardware combination provides a real time, hyper-precise, temperature-insensitive, closed-loop system which could benefit many optical communities, including EUV lithography, who require sub-nanometre bending control of the mirror form.

## Abstract 2

A closed-loop, adaptive deformable X-ray mirror system has been developed that enables synchrotron and XFEL beamlines to rapidly change and stabilize the size and shape of the X-ray beam. The optical surface is stabilized to the demand profile to <200 pm via voltage corrections applied autonomously to individual piezo actuators at 1 Hz, based on 20 kHz feedback from an array of interferometric sensors.

## 1. Introduction

A new generation of improved-quality X-ray optics are required to efficiently preserve the quality of ultra-intense X-ray beams created by new and upgraded low-emittance synchrotron storage rings and X-ray free-electron lasers (XFELs) (Cocco *et al.*, 2020 ▸, 2022 ▸; Seaberg *et al.*, 2019 ▸; Liu *et al.*, 2018 ▸; Cocco & Spiga, 2019 ▸). As the photon beams become smaller, brighter and more coherent, there is an increasing need to optimize the X-ray wavefront.

Total external reflection mirrors that use grazing incidence of a few milliradians are widely employed to focus or collimate X-ray beams. Achromaticity makes mirrors especially suited for spectroscopic energy scanning techniques. Highly reflective surfaces and large beam acceptance apertures also provide enhanced photon collection efficiency for micro- and nano-focusing compared with arrays of X-ray lenses. Recent technological advances enable X-ray experiments to be conducted in ever shorter timescales, hence many beamlines, particularly those dedicated to macro-molecular crystallography, now measure hundreds and sometimes thousands of samples per day.

Alongside fixed-curvature X-ray mirrors, most beamlines at synchrotron and XFEL facilities also use one or more ‘active’ optics for modulation and fine-tuning of the X-ray focus. Active optics employ a series of mechanical motors or piezo actuators to make localized or global changes to the mirror profile (Sutter *et al.*, 2022 ▸). Traditionally, such active X-ray optics are driven quasi-statically in open-loop mode. Changes to the mirror profile are only made every few hours or days. However, it is non-trivial to make frequent, accurate and stabilized large changes to active optics operating in open-loop. Mechanically bent mirrors are intrinsically not well suited for such a task since they are generally slow to respond, can suffer from backlash and premature ageing of mechanical parts with frequent activity, and are prone to over-heating when operated at high duty cycle in a high vacuum environment. In contrast, piezoelectric deformable bimorph mirrors (Signorato *et al.*, 1998 ▸) can respond almost instantly. They do not have mechanical components that wear out, and since they do not generate significant heat, they can by cycled indefinitely under vacuum.

Efficient usage of the photon flux demands frequent changes in the size and shape of the X-ray beam to optimally suit the dimensions of each crystal, or to illuminate different-sized regions of larger samples. Fixed-curvature X-ray mirrors that provide diffraction-limited performance are becoming commercially available. However, such optics do not readily provide the adaptability to correct X-ray wavefront errors caused by other non-ideal optics on the beamline, or time based dynamic variations caused by photon-beam heating, and they cannot be used to significantly expand the beam at the sample under test. Moderate beam size control at focus can be provided by slightly varying the incoming beam grazing angle, but this technique is prone to intensity-related striations and optical aberrations including coma. A bimorph mirror, operated with the closed-loop system demonstrated in this paper, and polished to diffraction-limit level (*e.g.* with residual figure errors below 1 nm r.m.s.), can provide X-ray beam size control with a significantly lesser level of striations and aberrations. The curvature of X-ray bimorph mirrors can drift by several percent over a few hours after a large voltage change (Alcock *et al.*, 2013 ▸). This potentially leads to corresponding transients in the size and shape of the reflected X-ray beam. Drift is caused by two factors: intrinsic piezo-electric creep of the actuators, and induced strain from the opto-mechanical holder and electrical cabling of the mirror which resist piezo-driven bending. The recent history of the mirror shape and applied voltages strongly influence the amplitude and direction of curvature drift and hysteresis. To reduce such drifts, a series of improvements were made, including the development of a new type of holder that minimizes mechanical strain, improved electrical connections to the mirror electrodes and deployment of an algorithm to correct piezo creep (Alcock *et al.*, 2019*a* ▸). This successfully reduced the time for stabilization of the reflected X-ray beam after large mirror curvature changes from tens of minutes to <30 s (Alcock *et al.*, 2019*b* ▸). However, this only worked for the specific transition between a well characterized start and end profile of the mirror. If a significantly different start or end shape was required, each transition had to be individually characterized, which is a time-consuming and impractical process if many such changes are required each day. Tests showed that it was not possible to empirically derive an optimized set of new transitions based on a linear combination of known transitions.

To achieve fast and precise control of the reflected X-ray beam, we previously developed an ‘adaptive’ closed-loop control system for a piezoelectric bimorph deformable mirror (Alcock *et al.*, 2023 ▸). First, X-ray beam diagnostics, such as speckle tracking (Wang *et al.*, 2015 ▸), are used to optimize the X-ray wavefront to one or more shapes by tuning the adaptive mirror. For example, a focused X-ray beam and a series of purposefully defocussed beams with minimal striations. Once optimization of the reflected beam is achieved, the user simply clicks on a button to record the shape of the mirror that achieves the desired X-ray wavefront. This process is repeated for all required wavefronts and a lookup table is built. In routine operation, the bimorph surface is measured at high-speed (up to 20 kHz) with sub-nanometre height sensitivity. The readout from multiple interferometric sensors is compared with the required height profile taken from the lookup table. Corrective voltages are autonomously calculated and iteratively applied at a refresh rate of ∼1 Hz using an HV-ADAPTOS power supply to rapidly switch and stabilize between the required mirror shapes and the X-ray wavefronts. There is no limit to the number of transitions to different shapes, only by the commissioning time invested in collecting them.

In this complementary paper, we present previously unpublished technical details about the hardware and software of this novel system, including extensive off-line experimental testing. It is hoped this will enable optical scientists and engineers to build and implement such devices for real time control and correction of the X-ray wavefront at synchrotron and XFEL facilities around the world.

## 2. Experimental setup

### 2.1. Hardware overview

**Figure 1:**
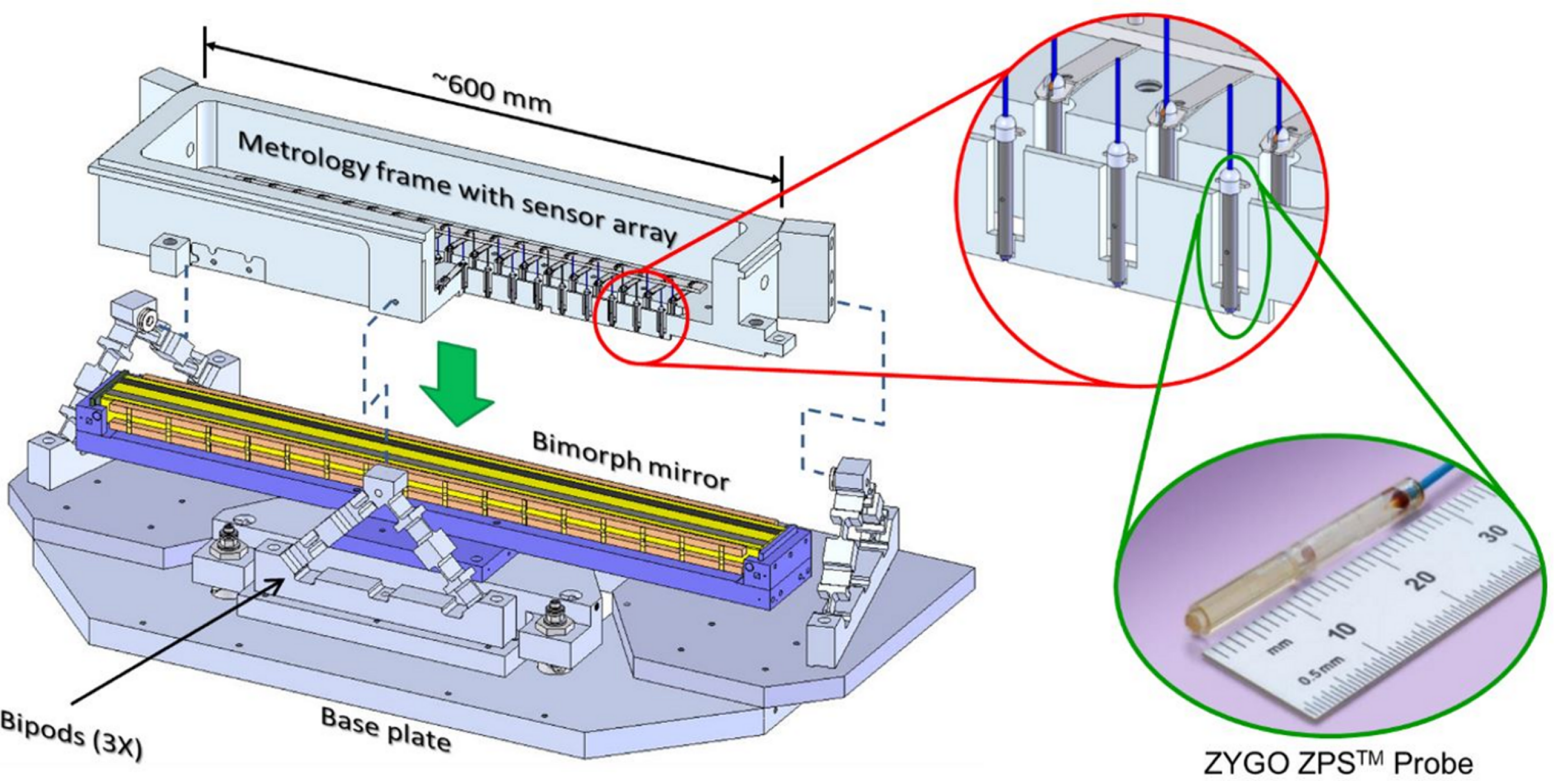
Optical components of the closed-loop, adaptive X-ray mirror system: piezo-deformable bimorph mirror and its opto-mechanical holder, and the ZPS multi-sensor fibre interferometer sensors mounted in an ultra-stable metrology frame.

As illustrated in Fig. 1 ▸, the main components of the closed-loop control, bimorph mirror system are: a piezoelectric bimorph deformable mirror, mounted in an opto-mechanical holder; a multi-sensor Zygo ZPS interferometer array, supported on an ultra-stable metrology frame; and a programmable HV-ADAPTOS high-voltage power supply.

#### 2.1.1. Bimorph deformable X-ray mirror

**Figure 2:**
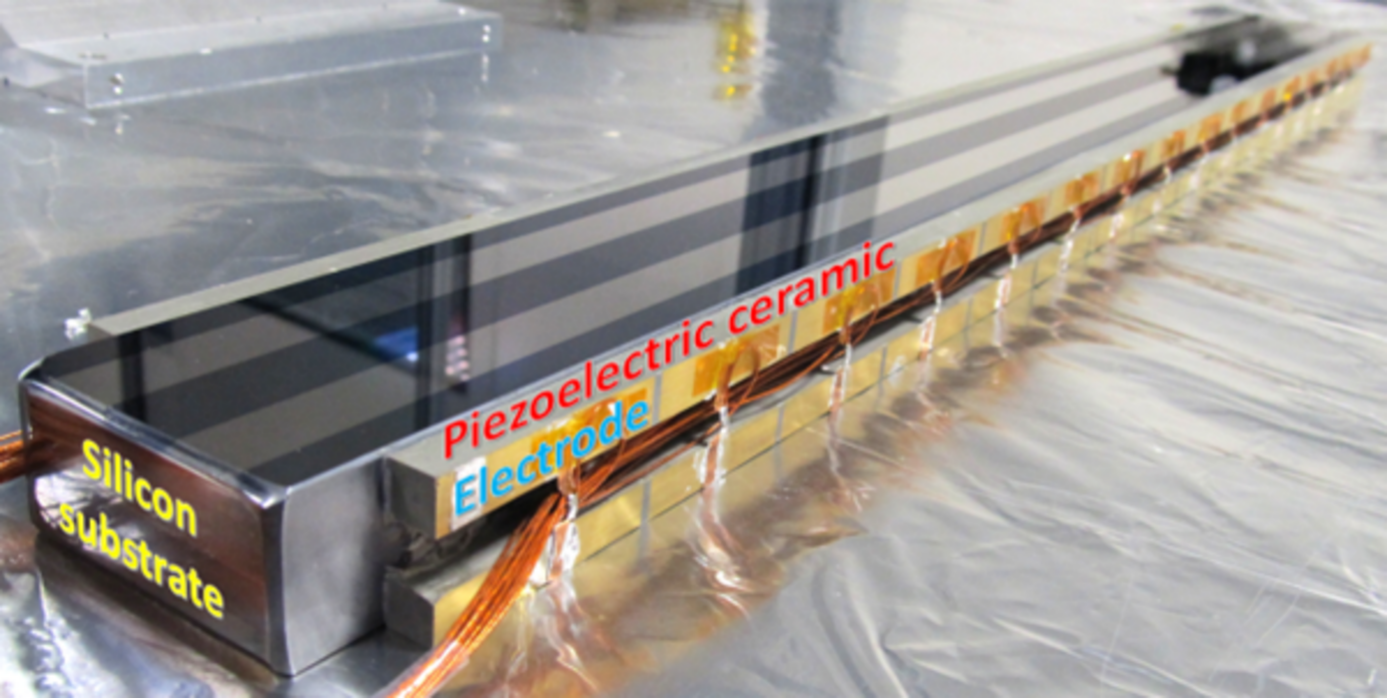
Second-generation bimorph deformable X-ray mirror (Thales-SESO patented), dismounted from its opto-mechanical holder, showing the piezoceramic bars bonded to the side faces of the silicon substrate. Distinct, gold-coloured metallic regions define the actuation region of each piezo electrode.

The bimorph mirror (see Fig. 2 ▸) is an early second-generation optic manufactured in 2013 by Thales-SESO, France (Alcock *et al.*, 2015 ▸). It is a 640 mm-long, single-crystal silicon substrate with 16 piezo electrodes. The optical surface was polished using ion beam figuring at Thales-SESO to an elliptical profile with a slope error of ∼500 nanoradians (nrad) root mean square (r.m.s.) and a height error of ∼5 nm r.m.s. which was a state-of-the-art result at that time. However, over the past decade optical polishing technology has significantly improved, and such optics are now commercially available with slope errors <100 nrad r.m.s. The mirror’s opto-mechanical holder was custom designed by S.RI.Tech, Italy (https://sri_tech@pec.it) to minimize the strain applied to the substrate (Alcock *et al.*, 2019*a* ▸) and to allow for reconfigurable mounting geometries (*i.e.* vertical or horizontal X-ray beam reflection). For this experiment, the mirror was mounted in a vertically deflecting, face-upwards configuration.

Zonal control of multi-electrode X-ray bimorph mirrors can also correct aberrations in the wavefront of the photon beam caused by imperfect optics located upstream or downstream (Wang *et al.*, 2015 ▸; Sawhney *et al.*, 2013 ▸). Higher-order, complex-surface profiles can also be applied to create reflected beams with a non-Gaussian cross-section, such as a ‘flat-top’ constant intensity profile (Sutter *et al.*, 2016 ▸), and even split it into multiple peaks (Alcock *et al.*, 2023 ▸). Structured light, rather than uniform illumination, is a field of renewed interest in many scientific disciplines (Forbes *et al.*, 2021 ▸).

#### 2.1.2. ZPS multi-sensor interferometric system and metrology frame

Displacement of the optical surface is simultaneously measured at multiple locations using an array of 2 rows of 19 interferometric, fibre based, ZPS sensors from Zygo, USA (Badami *et al.*, 2019 ▸). These devices record the absolute distance of the optical surface from the internal reference surface of each sensor, rather than the relative displacement. Unlike traditional interferometers, the ZPS sensors are immune to interruptions in the beam and can recover the absolute position of the mirror surface after power loss or beam obstruction. Each sensor is mounted in a deterministic exactly constrained manner in an aluminium metrology frame. In turn, this frame is supported in a kinematic decoupled fashion to provide an ultra-stable reference for measuring height changes of the mirror. Details of the mounting and the reasons for choosing aluminium can be found in Badami *et al.* (2019 ▸).

The key requirement for sub-nanometre monitoring is maintaining the rigid shape of the frame (and hence alignment/positioning of the ZPS sensor array) in the presence of temperature fluctuations. Slow changes in ambient temperature cause differential expansion between the frame and the structure to which it is mounted, as well as gradients within the frame. The former is minimized by the careful design of the frame mount, whereas the latter is handled through choosing an appropriate material for the frame. Overall, thermal changes cause expansion of the frame and the supporting structure, but do not change the shape of the frame. A flexure implementation of the classic Type-II Kelvin kinematic mount provides the requisite decoupling between the metrology frame and the baseplate (Smith & Chetwynd, 1994 ▸) while eliminating the hysteretic effects of friction typical of a contact-type kinematic mount.

**Figure 3:**
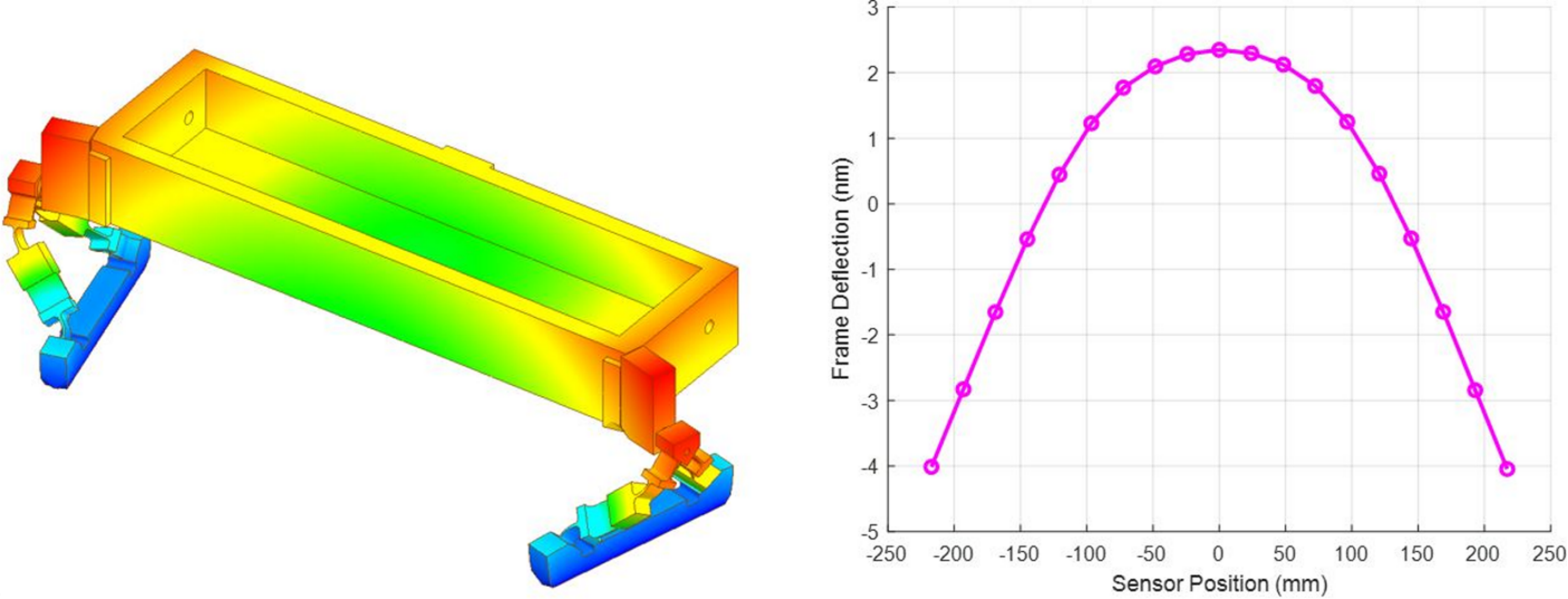
Flexing of bipods accommodates differential expansion of the metrology frame relative to the baseplate. (Left) Resultant 3D finite element analysis distortion of metrology frame caused by a 1°C temperature differential. (Right) Metrology frame deformation along the central tangential line of the metrology frame shows a 6.5 nm sagitta change for a 1°C temperature differential. Circles represent ZPS sensor locations.

Fig. 3 ▸ (left) shows finite element analysis modelling of how differential expansion between the baseplate and metrology frame is accommodated by flexing of the bipods. The worst-case scenario of a steady-state temperature difference between the baseplate and the metrology frame is modelled to derive the shape change sensitivity of the metrology frame. The base of the bipods is modelled as fixed, while the frame is modelled as having a uniform temperature 1°C higher than the baseplate. Fig. 3 ▸ (right) shows the resulting deformation of the frame along the array of ZPS sensors. This suggests a sagitta or ‘sag’ (depth at the centre of the mirror, relative to its two ends) sensitivity of ∼6.5 nm °C^−1^. This is in stark contrast to sag sensitivity of ∼200 nm °C^−1^ due to the coefficient of thermal expansion mismatch between the silicon mirror and the aluminium holder in which it is mounted (see Section 3.3). For representative temperature variations (0.1°C) in the laboratory where these tests were performed, the sag is predicted to change by only ∼0.65 nm. However, the thermal inertia of the system attenuates the impact of temperature fluctuations observed in the laboratory (∼45 min period and an amplitude of ∼0.1°C) and produces a sag change of ∼0.25 nm, which is less than 50% of the sag change predicted by the steady state result. Deformations of the metrology frame are thus much smaller than deformations of the bimorph mirror and can be considered negligible.

#### 2.1.3. HV-ADAPTOS high-voltage power supply for X-ray bimorph mirrors

The HV-ADAPTOS (https://www.caenels.com/products/hv-adaptos/) is a multi-channel high-voltage (HV) bipolar power supply developed to control, monitor and safely operate bimorph X-ray mirrors. The system has been continuously developed over the past 20 years, adding enhanced functionality, new communication protocols, and increased computing power and memory storage compared with the first units from the early 2000s (Cautero *et al.*, 2007 ▸). It is designed and distributed by S.RI.Tech, Italy (https://sri_tech@pec.it), and manufactured by CAEN, Italy: a specialist in electronics for scientific research. Voltages are applied to the bimorph mirror’s electrodes in a coordinated, parallel manner. The voltage slew rate can be as high as several hundred volts per second, thus allowing major curvature changes in a matter of seconds. Hardware-embedded features are designed to electrically protect the bimorph mirror, including passively controlled emergency output discharge, real time monitoring of current flow and spark detection, and parasitic current limitation. The system also includes proprietary software for compensation of piezo creep and hysteresis control. HV-ADAPTOS offers capabilities to rapidly communicate with external devices such as interferometers or X-ray wavefront sensors, and the beamline control system. It can be programmed to execute analysis scans (*i.e.* ‘beamlets’ slit scans across the mirror surface to acquire linear Hartmann tests) or to autonomously collect interaction matrix data. Once external data are collected, it can internally perform all calculations required by the adaptive correction and optimization algorithms, and null the errors in the mirror shape or X-ray beam profile relative to the specific user-defined target. This optimization process can be performed iteratively at up to ∼1 Hz, which ensures stability in time and overcomes non-linearity effects in the correction protocol.

### 2.2. Closed-loop architecture

**Figure 4:**
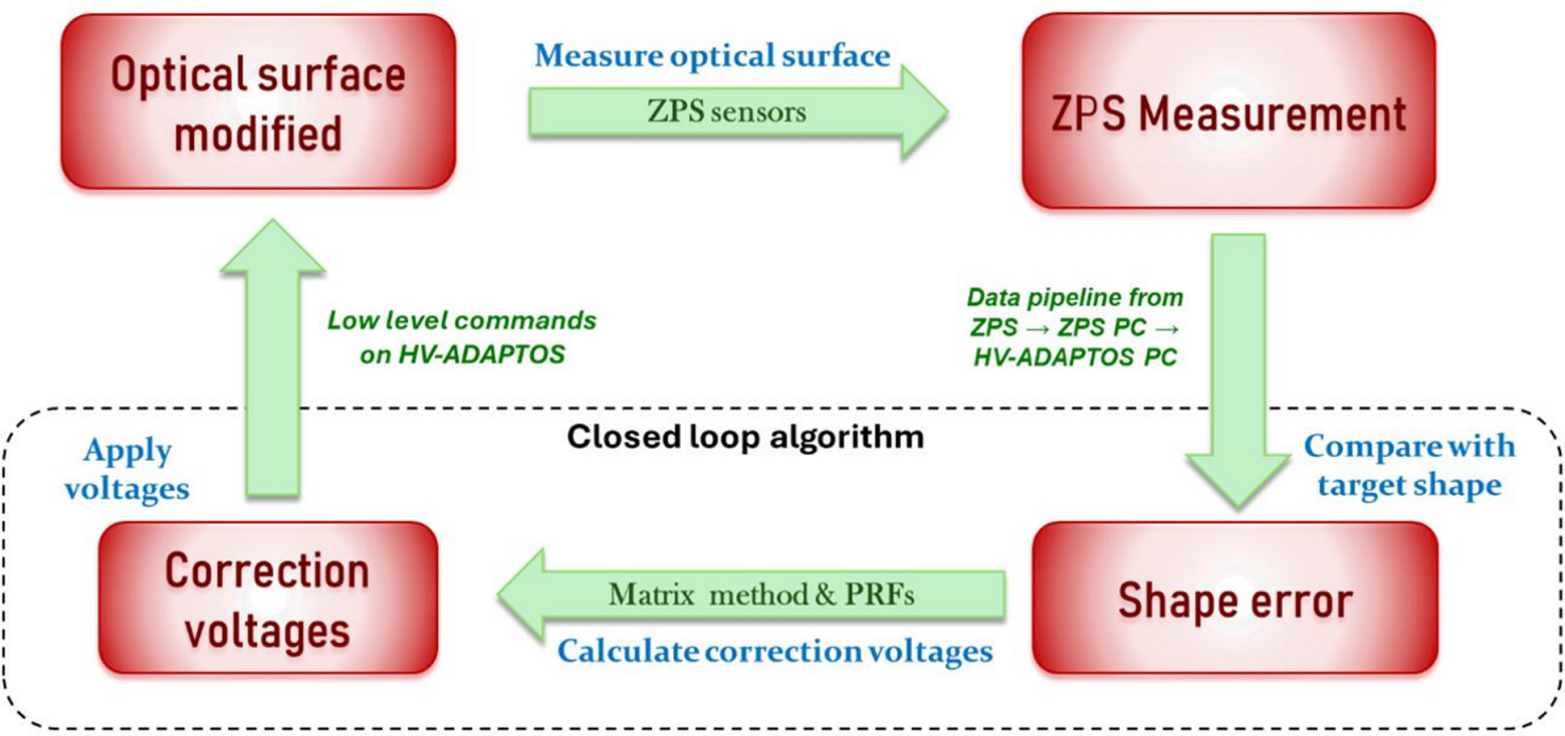
Functional diagram showing the correction mode of the closed-loop optical system. The target shape of the mirror can be changed at the user interface, or scripted to shift at specific times.

An overview of the control system is shown in Fig. 4 ▸. Interferometer data from the sensor array are sent at 20 kHz from the ZPS controller to a dedicated PC via sRIO high-speed protocol. Processed data are then sent from the PC to the HV-ADAPTOS power supply over an Ethernet TCP connection at a configurable rate. Calculations made on-board the power supply’s FPGA compare the current mirror shape to the target shape and compute the correction voltages using a proprietary algorithm. These voltages are then automatically applied to the respective bimorph electrodes to drive the mirror to the target shape. The closed-loop frequency is typically 1 Hz, but could be made faster if demanded by the end-users. Given that the maximum voltage of the bimorph is ±1500 V, and the maximum safe slew rate is 300 V s^−1^, even major changes in the reflected beam take just a few seconds to effect, while smaller corrections are virtually instantaneous (<1 s).

Closed-loop functionality enables two distinct modes of operation:

(1) Freezing the shape of the mirror (and the profile of the reflected X-ray beam). The interferometric feedback system continuously monitors for changes in the mirror shape, and voltage corrections are made to maintain the desired shape.

(2) Switching between user-defined surface profiles. This more advanced mode enables the user to rapidly switch and very precisely stabilize between specific pre-optimized profiles. A series of such changes can be made manually (via the user interface) or scripted to occur at specified times.

Regardless of the mode of operation, the mirror profile is held stable relative to the required profile with picometre level stability. The loop frequency of ∼1 Hz is sufficiently fast to enable fast shape changes (over a few seconds) and to negate the effect of mechanical/piezo creep or thermal drifts.

### 2.3. Metrology testing

**Figure 5:**
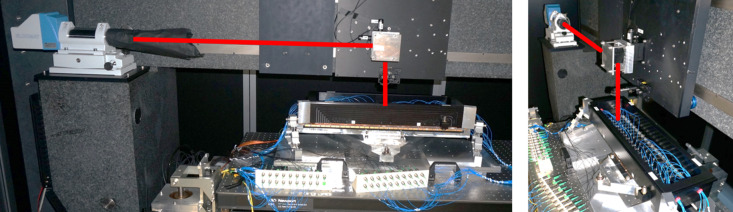
A cut-out in the metrology frame enables the autocollimator beam (red) of the Diamond-NOM slope profiler to measure the optical surface of the bimorph mirror, in synchrony with the on-board array of ZPS interferometers.

The closed-loop bimorph system was commissioned on the Diamond-NOM (Alcock *et al.*, 2010 ▸) in the Optics Metrology Lab (OML) at Diamond Light Source (Alcock *et al.*, 2016 ▸). The OML is a class 10000 (ISO7) cleanroom with excellent vibrational performance and temperature stability <0.1°C for prolonged periods of time. A thermal enclosure surrounding the Diamond-NOM further enhances stability to achieve temperature fluctuations of only a few millidegrees over many hours. A cut-out region in the metrology frame, between the two rows of ZPS sensors, provides another external metrology instrument with a line-of-sight view of the optical surface of the bimorph mirror (Badami *et al.*, 2019 ▸). In this manner, the ZPS system and the Diamond-NOM (or a Fizeau interferometer) can simultaneously monitor the bimorph mirror’s optical surface (see Fig. 5 ▸).

## 3. Results

### 3.1. Open-loop operation

**Figure 6:**
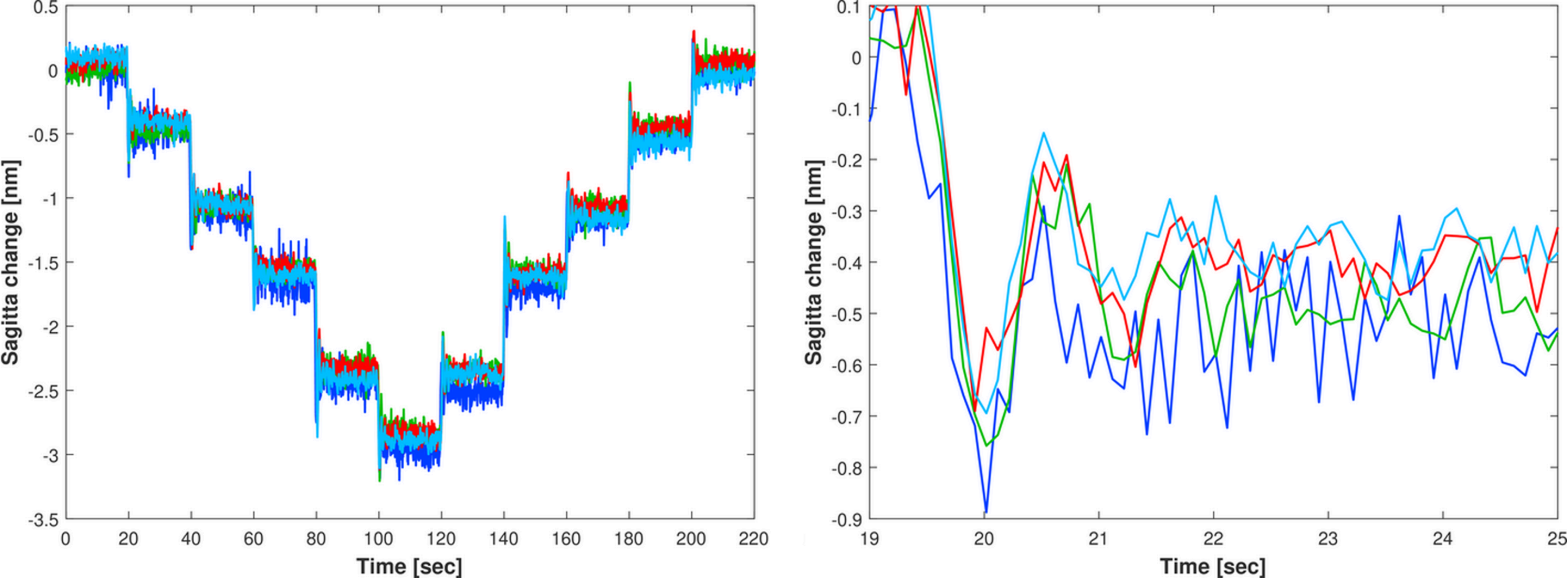
(Left) ZPS interferometer measurements of the sagitta/sag of the bimorph mirror as a series of four cycles of five bidirectional steps, each with the smallest possible voltage increments (0.1 V), were applied in open-loop by the HV-ADAPTOS to all piezo actuators to bend the mirror. The four cycles are superimposed to show the extreme repeatability in achieving sagitta steps as small as 500 pm. (Right) Zoom of the sagitta change of the mirror immediately after the first voltage step of 0.1 V shows a ‘ringing’ effect with a damped amplitude of ∼800–100 pm and a period of ∼1 s.

Each ZPS sensor is clamped into the metrology frame at a nominal stand-off distance of 3.5 mm from the optical surface. However, due to alignment tolerances, each sensor’s vertical offset within the metrology frame can vary by tens to hundreds of micrometres relative to its neighbours. These offsets can be calibrated relative to the optical surface by comparison with surface topography measurements by the Diamond-NOM. The location of peaks in the piezo response function profiles identifies specific locations on the optical surface viewed by different metrology instruments. Although the ZPS sensors are in two rows that straddle the central line of the mirror, the relative change in the shape of the mirror as different piezos are actuated is sufficient to fiducialize the in-plane positioning of the different metrology instruments to <1 mm on the optical surface. In turn, this helps to determine the vertical position of each ZPS sensor within the metrology frame. After calibration, a range of large voltages were applied to all piezo electrodes to bend the mirror, while simultaneously monitoring its surface with the Diamond-NOM and ZPS system. Both metrology instruments were demonstrated to be in good agreement (Alcock *et al.*, 2019*c* ▸). The Diamond-NOM takes several minutes to perform a single line scan along the mirror surface, and a Fizeau interferometer typically takes several seconds to record height data with sub-nanometre noise levels. Therefore, unlike the ZPS system, neither instrument can record fast dynamic changes in the bimorph mirror’s optical surface. In contrast, the left image in Fig. 6 ▸ shows the ZPS interferometer measurement of the dynamic behaviour of the mirror sagitta as a function of time, as four cycles of bidirectional steps of the smallest possible voltage increments of 0.1 V were sequentially applied, in open-loop, at 20 s intervals by the HV-ADAPTOS power supply. The cycles superimpose almost perfectly, demonstrating that the system can reliably make and measure 500 pm changes in the mirror sagitta with unprecedented precision. Note that it is even possible to observe that some steps are repeatedly larger (or smaller) by a few ångstroms than their neighbours. This can be attributed to the HV power supply applying slightly larger (or smaller) voltages for each specific step, according to its internal calibration and PID control. This is an unprecedented level of fast, precise atomic scale control and stabilization of a macroscopic optic of over 0.5 m in length.

The right image in Fig. 6 ▸ shows the ‘ringing’ effect (damped harmonic oscillation) of the mirror sagitta on the sub-nanometre scale immediately after a 0.1 V change. Oscillations have an ∼1 s period and an amplitude beginning at <1 nm peak-to-valley which quickly decays to <100 pm within 3 s. Since this effect is observed after each voltage jump, it can be attributed to the rapid acceleration impulse and subsequent stabilization of the mirror mass (several kilograms) within its holder. Phase agreement of the damped oscillations between successive cycles of voltage jumps excludes seismic or acoustic coupling of the system with random environmental noise.

**Figure 7:**
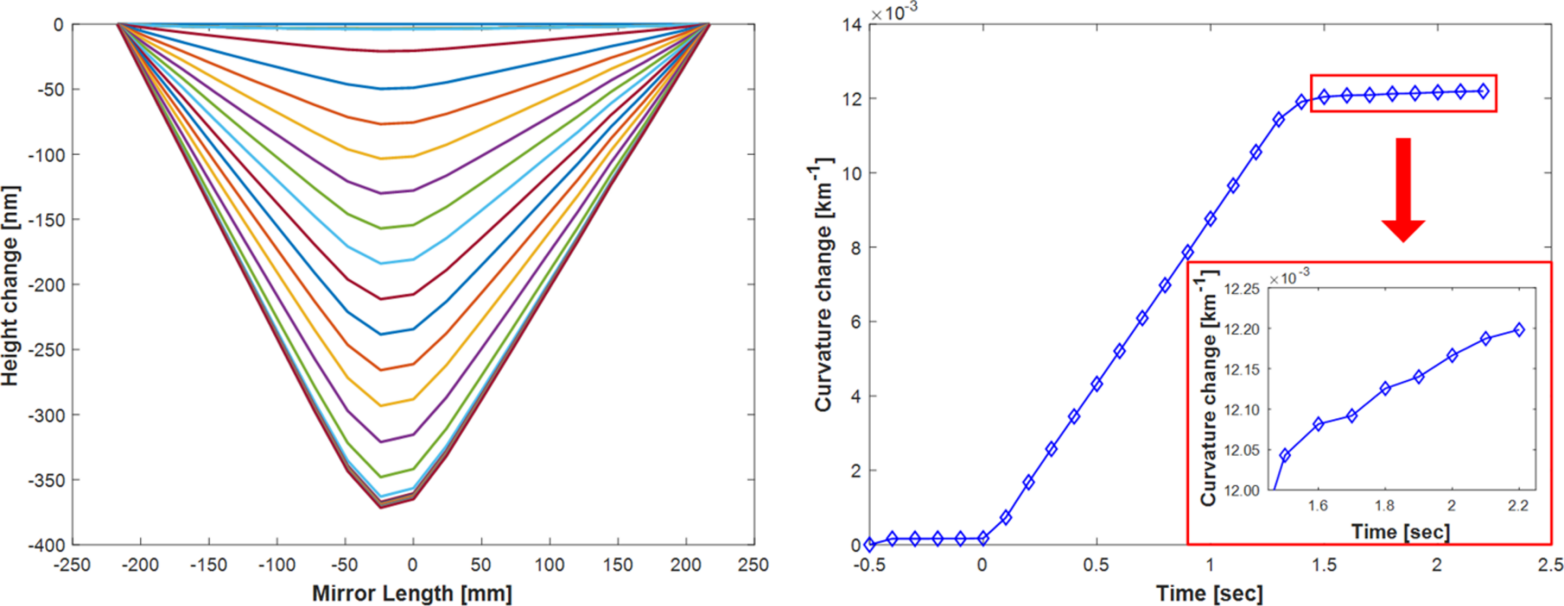
(Left) Dynamic evolution of the bimorph mirror’s surface profile, measured at 100 ms intervals over a few seconds, before, during and after applying a single piezo response impulse of 400 V to a central electrode. (Right) For the first 1.3 s after *T* = 0 s, voltage (applied at a slew rate of 300 V s^−1^) is linearly applied which causes a corresponding linear change in the mirror curvature. After this time, the surface stabilizes with small residual drifts in the curvature.

The bending response of a bimorph mirror is characterized by the so-called piezo response functions (PRFs), which are empirically measured by applying a known voltage to a specific piezo electrode and then observing how the mirror’s shape changes. This procedure is repeated for each independent electrode. Using the PRFs, an inverse-matrix method calculates the required voltage changes needed to bend the mirror from its current shape to any given new shape (Sutter *et al.*, 2011 ▸). Fig. 7 ▸ shows the dynamic response of the optical surface along the length of the bimorph mirror as it responds to a large voltage change of 400 V applied to a central piezo electrode. Each curve records the shape of the bimorph mirror’s optical surface at sequential intervals of 100 ms. Unlike the slow acquisition speed of the Diamond-NOM or a Fizeau interferometer, the ZPS sensors can reveal important temporal information, including how long it takes to apply voltages and bend the mirror, followed by the dynamic evolution of piezo creep and holder strain.

**Figure 8:**
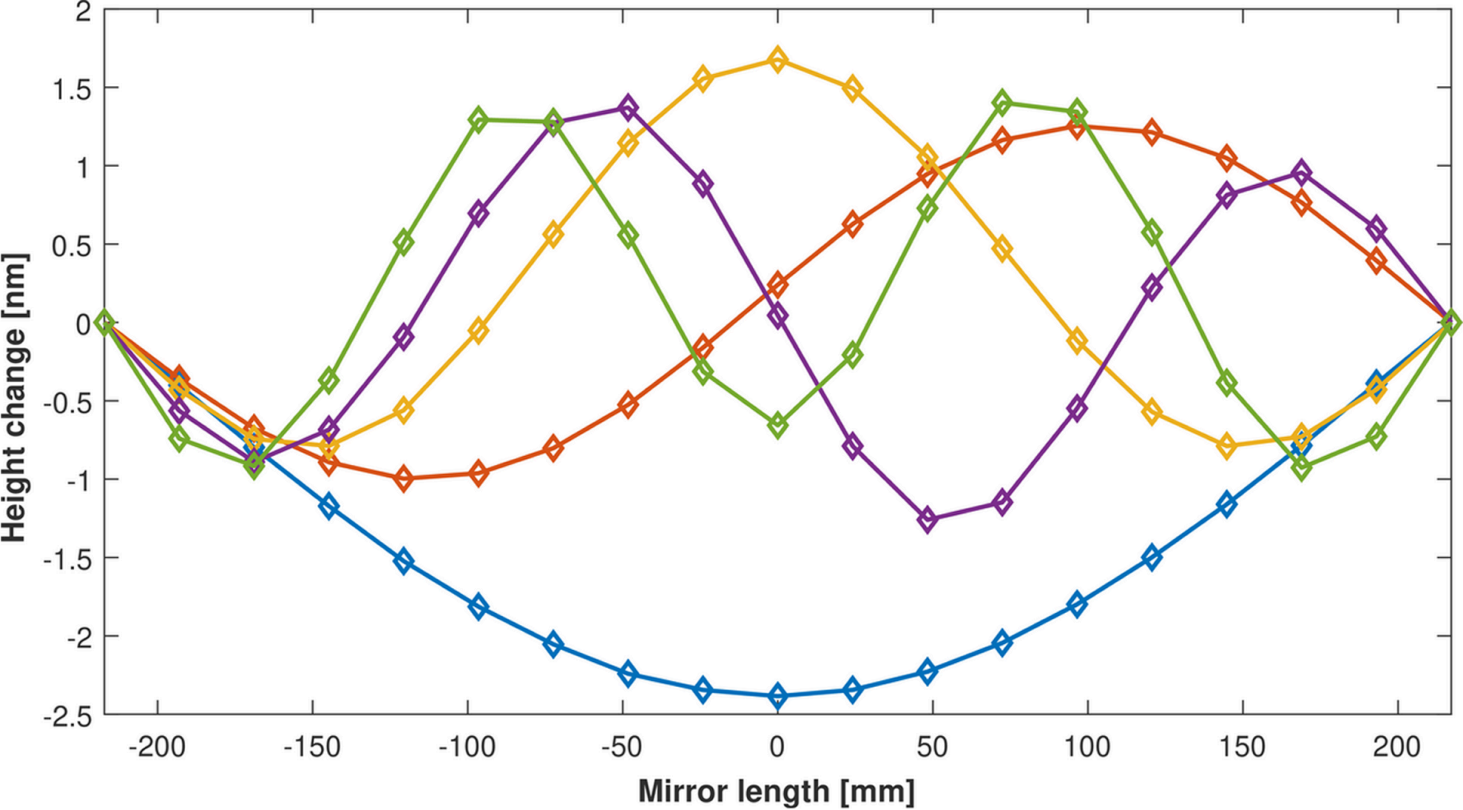
ZPS interferometer measurements of the optical surface, as voltages were applied every 20 s to sequentially add waves to the surface of the bimorph mirror (see the time-lapse video provided in the supporting information).

To further demonstrate the dynamic performance and sensitivity of the system, ZPS interferometer data were collected as the HV-ADAPTOS was instructed to modulate the bimorph mirror’s surface into a sequence of sine waves with nanometre-level amplitudes. The fundamental standing wave (half period) and overtones (1, 1.5, 2 or 2.5 periods) each had an amplitude of ∼2 nm and a dwell time of 20 s. ZPS data were averaged down to 10 Hz. Fig. 8 ▸ shows time-snapshots of the mirror as each wave was sequentially applied to the optical surface. A video showing the dynamic sequence of sine waves is provided in the supporting information.

### 3.2. Closed-loop operation: stabilization of bending drift

**Figure 9:**
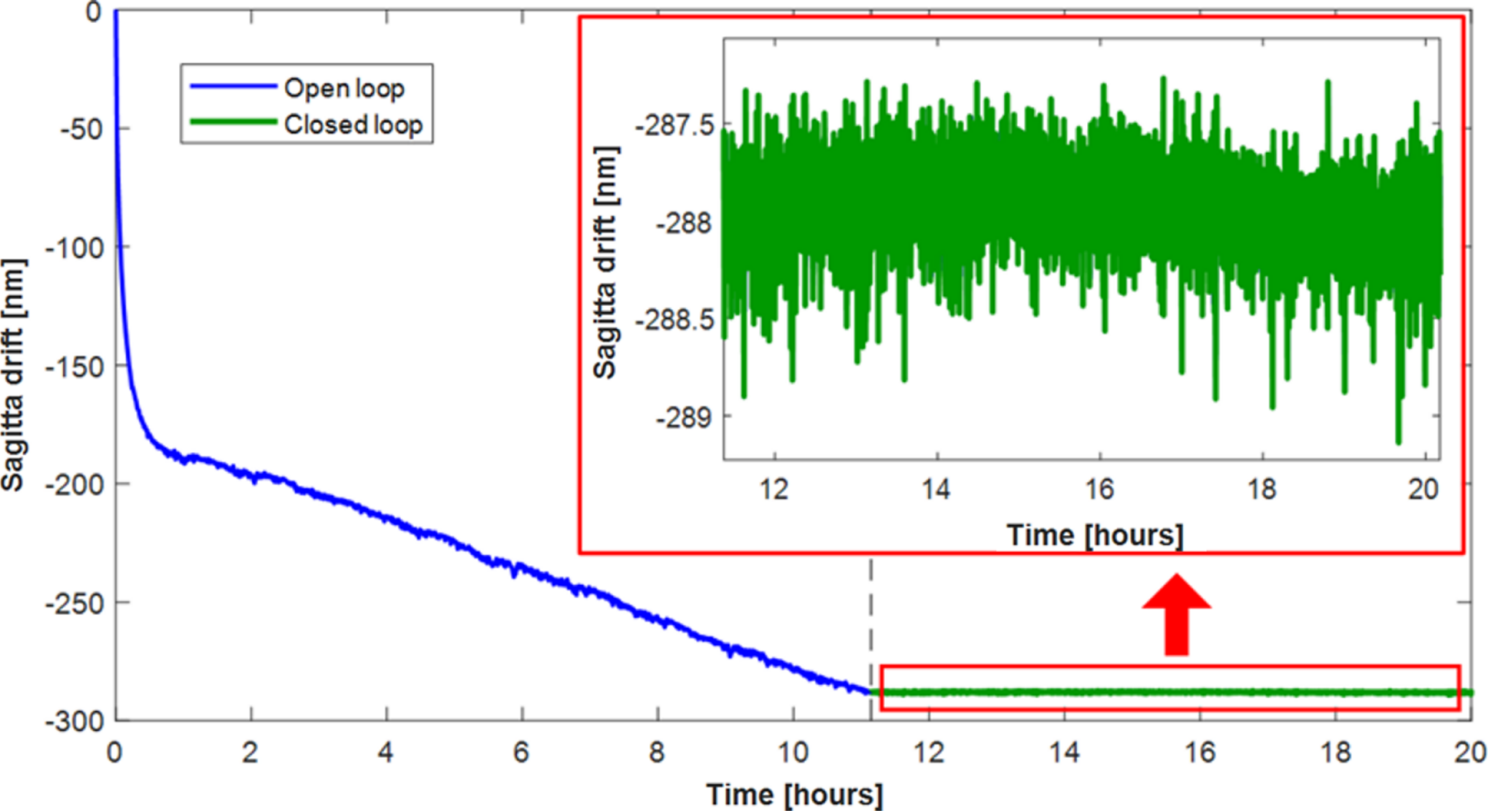
Long-term stability of the mirror sagitta after applying a large voltage change to the piezo electrodes. For the first ∼11 h, with the mirror operating in open-loop, the sagitta drifted by a few hundred nanometres (blue curve), corresponding to a radius change of ∼100 km. But after activating closed-loop (green curve), the sagitta was quickly stabilized and remained within 0.179 nm r.m.s. over the next ∼9 h.

To quantify the stability improvement provided by closed-loop operation, a large 1000 V pulse was applied to all electrodes to purposefully induce a sagitta change of ∼6 µm and subsequent drift in the mirror curvature (a combination of piezo creep and mechanical strain from the holder). With the mirror operating in open-loop, the ZPS interferometer sensors observed drifts of several hundred of nanometres (corresponding to a change in radius of ∼100 km) in the mirror sagitta over several hours (blue curve in Fig. 9 ▸). The drift equates to a few percent of the applied 6 µm sagitta change. After ∼11 h, closed-loop control was activated (green curve) which immediately stabilized the radius of curvature and continued to null any drifts throughout the remaining 9 h of the experiment. Variation of only 0.179 nm r.m.s. was observed in sagitta drift during ∼9 h of closed-loop operation (inset image in Fig. 9 ▸).

**Figure 10:**
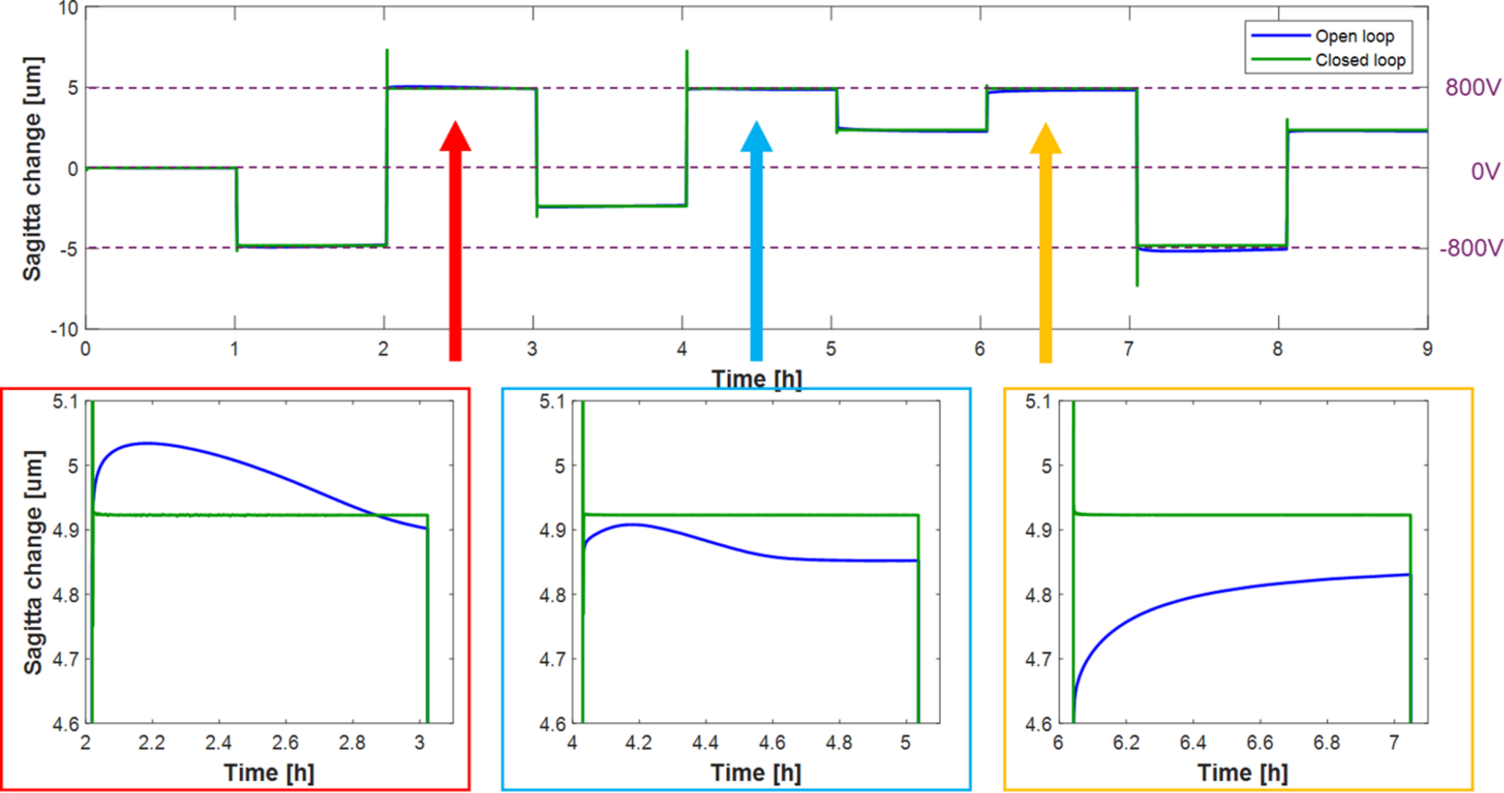
(Upper plot) Sagitta change of the bimorph mirror when a series of large voltage jumps (−800, −400, 0, 400, 800 V) were sequentially applied to change the curvature at hourly intervals in either open- (blue curve) or closed-loop (green curve). (Lower plots) Zoom in on three regions of the upper plot, all corresponding to the same applied voltage. Closed-loop successfully stabilizes the mirror radius, whereas unpredictable drifts of up to 150 nm occur in open-loop (due to piezo creep and strain from the holder).

The effectiveness of closed-loop control was further demonstrated by applying a series of large (multiples of 400 V) voltage changes to all piezos every hour and monitoring the change in the mirror profile using the ZPS sensor array. Fig. 10 ▸ shows the measured sagitta change for open-loop (blue curve) and closed-loop (green curve). Zooming in on individual steps demonstrates non-deterministic drift of the mirror shape in open-loop (a maximum of ∼3% of the curvature change). The direction and amplitude of drift are complicated and depend on multiple factors, including recent history of voltages applied to the mirror and environmental stability. Conversely, in closed-loop, the mirror quickly and reliably returned to the same curvature and remained stable indefinitely.

**Figure 11:**
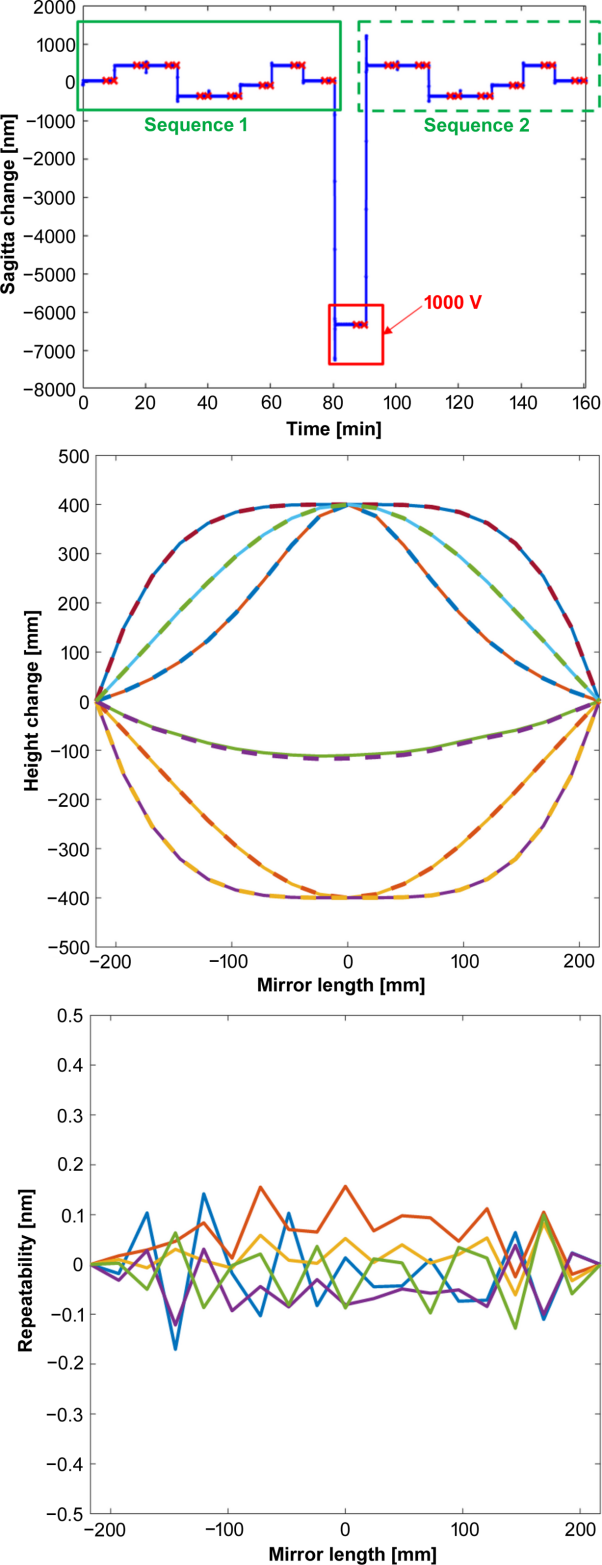
(Upper image) Timeline of the mirror sagitta, showing two identical sequences of eight Gaussian, Lorentzian or polynomial shapes applied to the mirror. The first sequence begins with the mirror at a stable shape of zero volts, whereas the second sequence starts after a 1000 V intentional ‘kick’ to the mirror. (Middle image) Height change of mirror shapes after the 1000 V impulse relative to the 0 V state. The curves are indistinguishable. (Lower image) Calculated difference between corresponding shapes in the two sets of measurements in closed-loop. Repeatability errors are ∼30 picometres r.m.s., reaching single-atom levels.

To investigate closed-loop operation for more sophisticated, non-cylindrical surface profiles, Fig. 11 ▸ shows two runs of a sequence consisting of five different concave or convex surface profiles (Gaussian, Lorentzian or polynomial) applied to the mirror, each for a duration of 10 min. The first sequence began with the mirror in an ultra-stable state (0 V applied to all piezos for several hours). However, before the second series began, a large +1000 V impulse (change in sagitta of 6 µm) was intentionally applied to all electrodes to purposefully induce large curvature drifts. In open-loop, this would significantly affect repeatability of the applied shapes for the second cycle. But with the mirror running in closed-loop, the difference between each corresponding shape in the two sequences was ∼30 pm r.m.s. and ∼200 pm PV, hence at the atomic level. This demonstrates that the closed-loop algorithm will accurately and quickly return the mirror to any given shape with unprecedented extreme precision, regardless of recent bending history.

### 3.3. Closed-loop operation: nulling optical deformation caused by thermal expansion

The above tests were performed inside the thermal enclosure of the Diamond-NOM, which provides millidegree temperature stability. However, under beamline conditions, the temperature may vary more drastically. Even within a UHV chamber, material temperatures can vary by a few fractions-of-a-degree, although with a long time constant if mirror positioners are built according to good engineering practice. The bimorph mirror construction is symmetric and hence is intrinsically insensitive to temperature changes. However, if friction pins the silicon substrate to the aluminium holder, then differential thermal expansion could cause a parasitic bending force. To quantify this effect, the system was removed from the Diamond-NOM’s thermal enclosure and placed into the main area of the laboratory, where the temperature varies sinusoidally over ∼1 h by ∼0.1°C PV. The bipod flexure design of the metrology frame holding the ZPS sensors is designed such that temperature changes only lead to rigid-body motions of the frame and do not cause appreciable bending or twisting. In this manner, the metrology frame provides a temperature-invariant frame of reference from which to monitor the optical surface.

**Figure 12:**
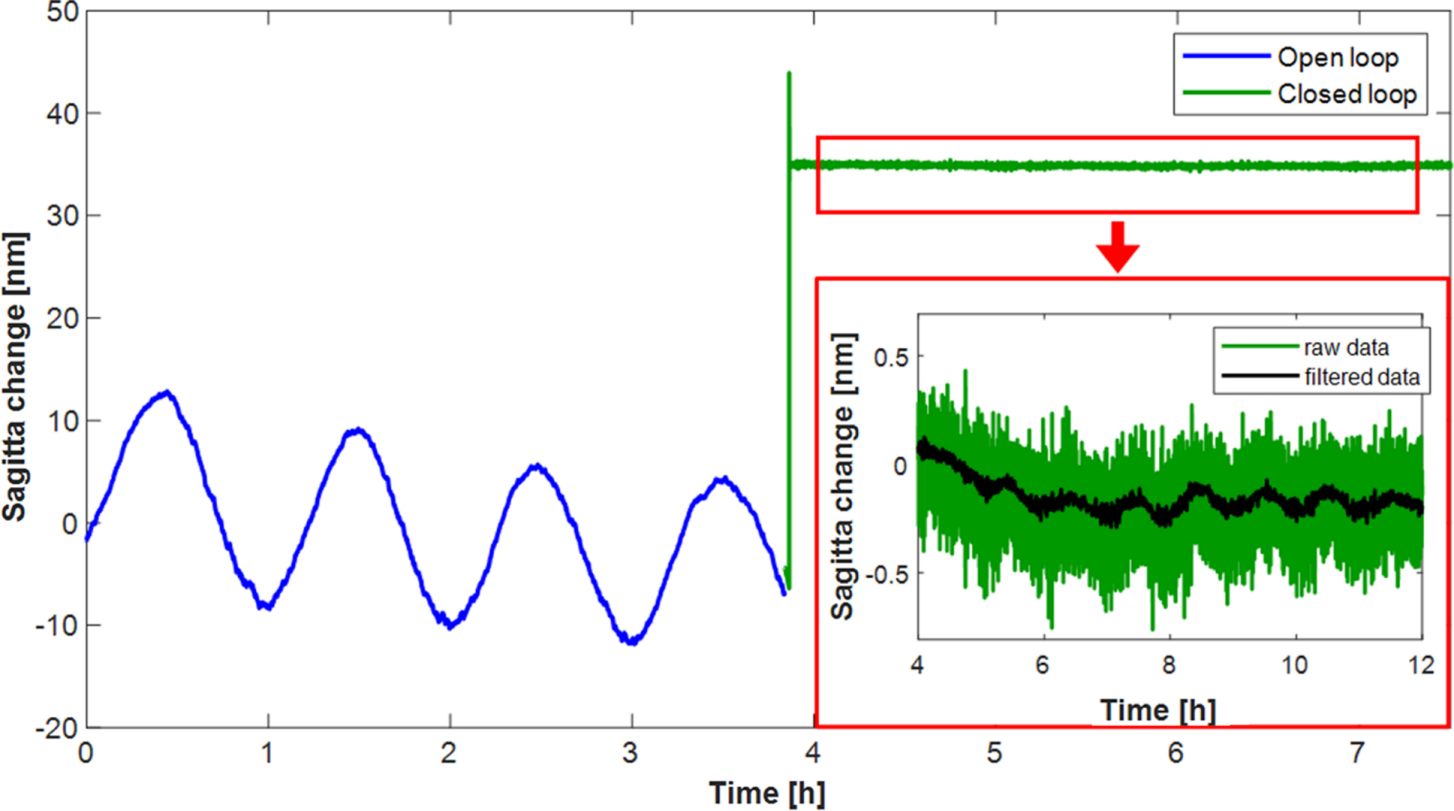
In an environment where the temperature varies sinusoidally with an amplitude of ∼0.1°C with a ∼1 h periodicity, the curvature of the mirror in open-loop (blue curve) follows a similar trend (most likely caused by relative thermal expansion between the dissimilar materials of the substrate and its holder). However, once closed-loop is activated (green curve), the mirror shape is instantly stabilized. The inset image shows a zoom of the closed-loop stability (filtered data are a moving average with a window of 101 data points) and a much weaker correlation between temperature and the mirror curvature.

With the mirror operating in open-loop, a clear correlation between the sagitta of the mirror (blue curve in Fig. 12 ▸) and the sinusoidal variation of the laboarotory temperature was observed. A 0.1°C temperature change caused a radius change of ∼1080 km, equivalent to a sagitta change of 20 nm. Given the thickness of the substrate (∼20 mm), this change in sagitta corresponds to only one part in a million. After ∼4 h, closed-loop control was initiated, which produced an immediate nulling of the thermally induced radius changes, as seen in the green curve in Fig. 12 ▸. In closed-loop, the thermally induced sagitta change was almost 70× smaller than in open-loop mode: the radius changed by only ∼71900 km (more than 10× the Earth’s radius), corresponding to a sagitta change of ∼100 pm for a 0.1°C change. This demonstrates the extreme robustness of closed-loop control for the mirror in a dynamic temperature environment, including thermal fluctuations on the beamline or a photon-induced heat load.

### 3.4. Long-term structural stability of the bimorph mirror

**Figure 13:**
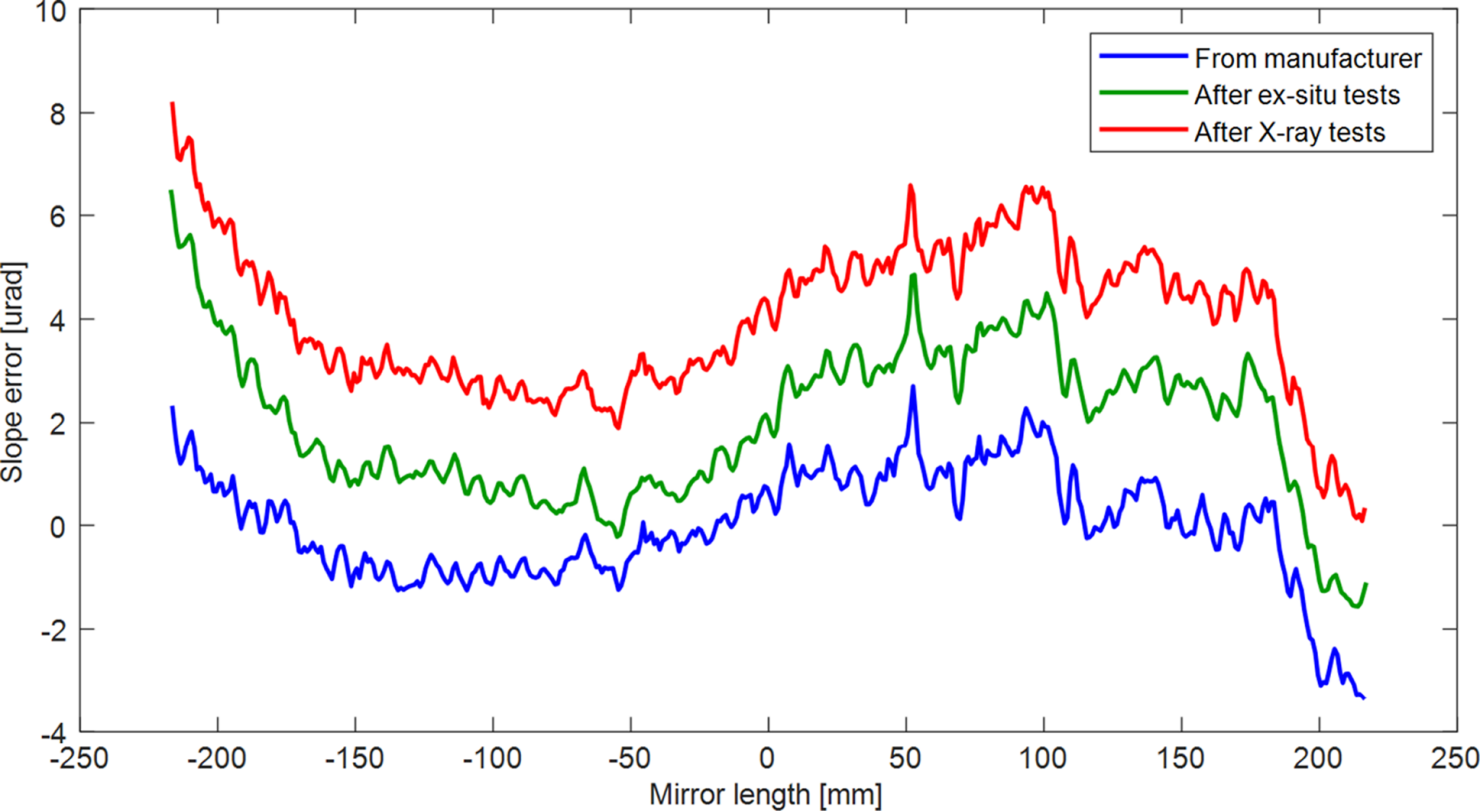
Slope error of the bimorph mirror: as received from the manufacturer in 2013 (blue curve), after >1 million cycles of closed-loop operation (green curve) and after X-ray tests (red curve). No change is observed in the optical surface.

The bimorph mirror used in this study is a relatively obsolete device, more than 10 years old, and was not originally designed for frequent, high-speed changes in shape. It is reasonable to question whether such a mirror can be cycled at 1 Hz for prolonged periods of time without becoming damaged. During development and commissioning of the closed-loop system, voltages were applied to the bimorph mirror every few seconds for several months. A conservative estimate is that >1 million voltage cycles were applied. We believed this to be many orders of magnitude more than any other synchrotron bimorph X-ray mirror in use today. Additionally, the voltage slew rate of 300 V ^−1^ is >10 times higher than traditionally applied to quasi-static, open-loop bimorph mirrors. The blue curve in Fig. 13 ▸ shows the slope error of the bimorph mirror as received from the manufacturer in 2013. After many months of *ex situ* metrology testing, with more than a million voltage cycles, no change was observed in the optical surface (green curve), confirming that the ‘junction effect’ had not occurred (sharp spikes in the optical surface at the glued interface between adjacent blocks of piezo ceramics). Similarly, no damage was observed after X-ray tests (red curve).

**Figure 14:**
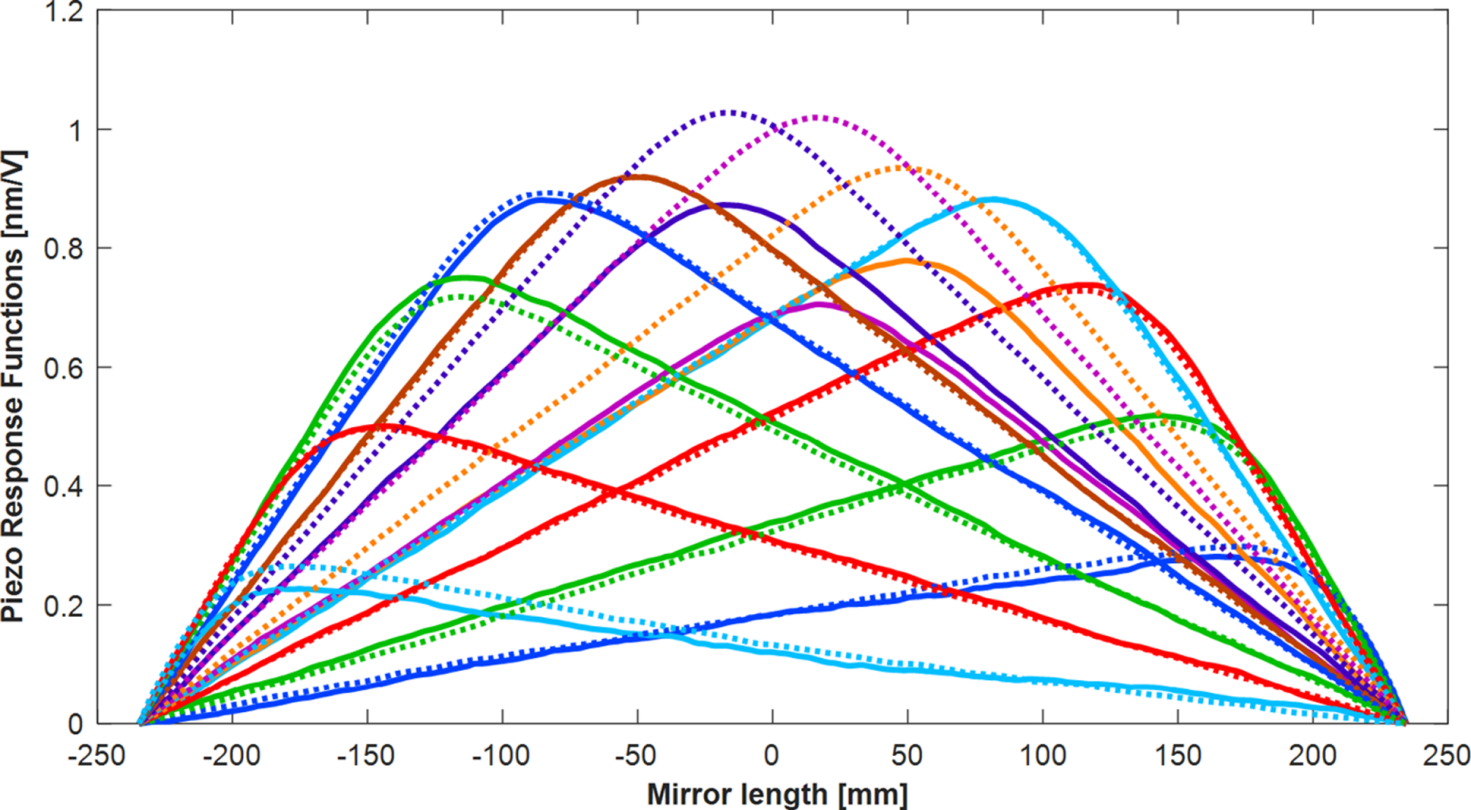
Piezo response functions show how each actuators responds to an applied voltage. Comparison between 2013 (continuous lines) and 2023 (dotted lines) shows that the piezos are still as strong as when the mirror was delivered to Diamond. In fact, three of the central piezo actuators have increased in strength, which is hypothesized to be caused by desiccation of moisture absorbed in the piezo ceramics.

Secondly, we investigated whether the bending strength of the piezo actuators diminished over time. This could occur if bonding of the glued interfaces between the piezo ceramic bars and the side faces of the silicon substrate were weakened by the rapid and frequent voltage changes. Fig. 14 ▸ shows Diamond-NOM scans of the piezo response functions as received from the manufacturer in 2013 (solid lines) and after closed-loop operation (dotted lines). No decrease in piezo strength was observed. In fact, three of the central electrodes (deposited on the same piezo ceramic bar) even slightly increased in strength. Amplitude variations in piezo responses have been observed previously at Diamond during long-term, *ex situ* tests of second-generation bimorph mirrors: some, but not all PRFs, gradually became slightly weaker over time. It is hypothesized that this effect is caused by absorption of moisture in some (but not all) piezo ceramics when HVs are applied to the mirror under atmospheric conditions. This effect was found to be reversed once the mirror was installed under vacuum. It is likely that the absorbed humidity is outgassed, thereby returning the weakened piezos to full strength.

### 3.5. X-ray exposure of ZPS sensors

After several days of exposure to monochromatic and white-beam (broadband) X-rays from the bending magnet source of the B16 Test beamline at Diamond Light Source (Alcock *et al.*, 2023 ▸), the ZPS sensors were observed to be visibly discoloured. However, optical tests after X-ray illumination confirmed that all sensors were fully operational, with only a marginal decrease in signal strength. This can be explained since the colour changes are transparent to the IR light used by the ZPS sensors. Despite these promising results, more rigorous experiments are required to quantify the point at which irreversible damage occurs as a function of X-ray wavelength and dosage.

## 4. Conclusions

We have developed an adaptive X-ray mirror system operating in closed-loop, based on fast metrology feedback from an array of ZPS interferometric sensors. The optical profile of the mirror is continuously monitored at 20 kHz, with voltage corrections made to individual piezo actuators at 1 Hz. Closed-loop control enables picometre-level stabilization of the optical surface. The user can repeatably apply and stabilize freeform target profiles to the mirror, regardless of recent history of bending or voltage changes.

The design of the metrology frame holding the ZPS interferometer sensors makes it insensitive to thermal changes. This innovation enables closed-loop operation to vastly reduce variations in the mirror shape caused by thermal fluctuations in the environment, or to nullify an X-ray induced heat-bump. Extensive tests before and after X-ray operations demonstrated that the mirror can mechanically withstand more than a million voltage cycles without apparent damage. Similarly, the ZPS sensors continued to operate correctly even after several days of proximity exposure to the high-intensity broadband X-rays from a bending magnet source.

The ultimate focusing performance of the old adaptive mirror used for this study is limited by the mid- and high-spatial frequency polishing errors on the substrate. If modern-day super-polishing techniques were applied to this mirror, the system would be ideal for ultra-precise and fast wavefront control of reflected X-ray beams. The closed-loop refresh rate is currently set by high-level software commands sent to the HV-ADAPTOS. However, with enhanced low-level control of the electrical hardware, there is scope to increase this rate to tens or hundreds of hertz.

Although this adaptive optics system was designed for synchrotron X-ray and XFEL applications, we believe that the ability to control an optical profile to <200 pm could be highly beneficial to many optical communities such as EUV lithography who demand ultra-stable diffraction-limited focusing and defocussing performance.

## Supplementary Material

Video file for Fig. 8. DOI: 10.1107/S1600577524011007/mo5292sup1.avi
